# Population-Level Metrics of Trophic Structure Based on Stable Isotopes and Their Application to Invasion Ecology

**DOI:** 10.1371/journal.pone.0031757

**Published:** 2012-02-21

**Authors:** Michelle C. Jackson, Ian Donohue, Andrew L. Jackson, J. Robert Britton, David M. Harper, Jonathan Grey

**Affiliations:** 1 School of Biological and Chemical Sciences, Queen Mary University of London, London, England; 2 School of Natural Sciences, Trinity College Dublin, Dublin, Ireland; 3 Trinity Centre for Biodiversity Research, Trinity College Dublin, Dublin, Ireland; 4 Centre for Conservation Ecology and Environmental Change, Bournemouth University, Bournemouth, England; 5 Department of Biology, University of Leicester, Leicester, England; 6 Department of Physiological Ecology, Max Planck Institute for Limnology, Plön, Germany; National Institute of Water & Atmospheric Research, New Zealand

## Abstract

Biological invasions are a significant driver of human-induced global change and many ecosystems sustain sympatric invaders. Interactions occurring among these invaders have important implications for ecosystem structure and functioning, yet they are poorly understood. Here we apply newly developed metrics derived from stable isotope data to provide quantitative measures of trophic diversity within populations or species. We then use these to test the hypothesis that sympatric invaders belonging to the same functional feeding group occupy a smaller isotopic niche than their allopatric counterparts. Two introduced, globally important, benthic omnivores, Louisiana swamp crayfish (*Procambarus clarkii*) and carp (*Cyprinus carpio*), are sympatric in Lake Naivasha, Kenya. We applied our metrics to an 8-year data set encompassing the establishment of carp in the lake. We found a strong asymmetric interaction between the two invasive populations, as indicated by inverse correlations between carp abundance and measures of crayfish trophic diversity. Lack of isotopic niche overlap between carp and crayfish in the majority of years indicated a predominantly indirect interaction. We suggest that carp-induced habitat alteration reduced the diversity of crayfish prey, resulting in a reduction in the dietary niche of crayfish. Stable isotopes provide an integrated signal of diet over space and time, offering an appropriate scale for the study of population niches, but few isotope studies have retained the often insightful information revealed by variability among individuals in isotope values. Our population metrics incorporate such variation, are robust to the vagaries of sample size and are a useful additional tool to reveal subtle dietary interactions among species. Although we have demonstrated their applicability specifically using a detailed temporal dataset of species invasion in a lake, they have a wide array of potential ecological applications.

## Introduction

The pace of global environmental change has increased substantially in the last hundred years due to new environmental pressures as a result of human activity [1]. Human-mediated introductions of alien species are one of the most widespread and damaging of these pressures and, although some introductions may have neutral impacts on ecosystems, others have adverse effects on both assemblage composition and ecosystem functioning [2]. The possibility of waves of successful invasive species facilitating establishment of further invaders by disrupting ecosystem structure and functioning has given rise to the Invasion Meltdown Model [3]–[5]. Many ecosystems now sustain several sympatric invasive species and yet their interspecific interactions are generally poorly understood [6], [7]. Knowledge of these interactions is key to understanding, and thus predicting, changes in trophic structure and assemblage composition instigated by sympatric and successive invaders. Integral to the Invasion Meltdown Model is that sympatric invaders promote the survival and potentially exacerbate the adverse effects of others [3]. Conversely, sympatric invasive species belonging to the same functional feeding group may exhibit a degree of dietary overlap potentially leading to strong interspecific competition when resources are limiting [7].

Stable isotope analysis is a contemporary tool to study the food web consequences of species invasions [8]–[10]. In ecological studies, the most commonly used naturally occurring stable isotope ratios are ^15^N:^14^N and ^13^C:^12^C which can be used to create ‘maps’ of food webs and hence, infer putative energy sources, trophic linkage and trophic position [11]. Stable isotope metrics enable the quantification of trophic structure at the community-level [12] and individual variation of δ^13^C and δ^15^N within populations can provide useful information on population trophic ecology [13], [14]. Recent developments in isotope ecology have provided statistical frameworks for examining variation among the isotope values of defined groups [15], [16]. Further, Layman et al. [14] described how the convex hull area occupied by a species in δ^13^C-δ^15^N isotopic space represents trophic diversity and can, therefore, be used as a quantitative indication of niche space. Jackson et al. [17] extended these methods and strengthened their ability to cope with disparities in sample size.

Classic theory [18], [19] suggests that a given species will occupy a larger realised niche in the absence of interspecific competition and yet sympatric species can only have a limited degree of resource use overlap before competitive exclusion occurs [20]. Consequently, there should be an inverse relationship between the isotopic niche space occupied by a species and the degree of interspecific competition it experiences. Thus, sympatric invaders belonging to the same functional feeding group would be expected to exhibit a smaller isotopic niche than their allopatric counterparts. However, a credible alternative hypothesis might be that increased competition for resources results in a more varied diet in order to maintain energy requirements and hence a larger isotopic niche [21]. We sought to test these hypotheses by applying stable isotope metrics at the population-level, as a logical progression of the metrics proposed by Layman et al. [12] which provide quantitative measures of the trophic structure of entire communities [12].

We chose Lake Naivasha in Kenya to test our hypotheses as it is a large (∼150 km^2^) natural freshwater ecosystem that has been subject to numerous species introductions over at least an 80 year period [22]. Britton et al. [23] reported that five of the six fish species currently present are non-indigenous and the lake also harbours several alien plant species, along with the globally widespread invasive Louisiana red swamp crayfish, *Procambarus clarkii*. Inverse correlations between native submerged macrophyte (*Potamogeton schweinfurthii*, *P. pectinatus*, *P. octandrus* and *Najas pectinata*) density and crayfish abundance, resulting in a dynamic, cyclic trend of crayfish and aquatic plant biomass, have led to the suggestion that the crayfish was a keystone species in the lake [22]. The most recent introduction to the lake in 1998 was of another globally widespread invasive species, carp, *Cyprinus carpio*, which has dominated the commercial fishery since 2003 and can contribute up to 98% of catches [24]. Elsewhere, invasive carp tends to be a keystone species, having profound effects on species composition and trophic linkages [25]. Crayfish and carp belong to the same functional feeding group as both are relatively large benthic omnivores. They might, therefore, be expected to interact strongly as a result of dietary overlap. However, the size discrepancy in adults may influence their preferred food source resulting in distinct dietary niches. Indeed, large carp attain sufficient gape size to ingest crayfish as prey [23].

We used stable isotope data and concurrent ecological data spanning 8 years (2001 to 2008) to examine the trophic interactions occurring between the most recent invader, carp, and the previously established invader, crayfish. We used new, robust stable isotope metrics applicable to individual populations [17] to investigate fluctuations in trophic diversity and quantify shifts in each species' isotopic niche. Specifically, we tested the hypothesis that carp and crayfish would express dietary overlap because they belong to the same functional feeding group and eventually that carp would suppress and/or displace the isotopic niche of crayfish due to competitive superiority.

## Methods

### Ethics statement

All animal work was conducted in accordance to national and international guidelines to minimise discomfort to animals (Schedule 1 of the Animals [scientific procedures] Act, 1986). Since there were no regulated procedures involved, the Max Planck Institute for Limnology board reviewing the project declared there was no requirement for ethics approval. All necessary permits were obtained for the described field studies from the National Council for Science and Technology, Kenya: NCST 5/002/R/020-D (formerly OP/13/001/12C46).

### Sampling and laboratory analyses

Lake Naivasha was sampled annually between 2001 and 2008 over 15-day periods in July. The same ten sites were sampled around the lake each year for carp, crayfish, macrophytes, sediment, plant debris and benthic invertebrates. Adult crayfish abundance was quantified using crayfish traps baited with dead fish. After 1–2 h, traps were lifted, all crayfish were counted and catch per unit effort (CPUE; number of crayfish per trap per hour) calculated. Abundance of carp was estimated using the CPUE from multi-panel gill-nets. Gill nets were lifted after 2–5 h of fishing and all carp were removed for counting, placed in containers and euthanised using an overdose of anaesthetic (MS-222 or benzocaine). Alternative fish sampling techniques to gill netting were not available; seine netting could not be used effectively due to poor shoreline access and the danger of disturbing hippopotami, and electric fishing equipment was not available in that area of Kenya. Submerged plants were sampled by dragging a double-headed rake along the sediment in three 25 m transects at each of the 10 sites, and the quantity of living plant material was estimated on a relative five-point scale. Dominant benthic invertebrates (primarily chironomids and oligochaetes), sediment, plant debris and floating macrophytes (*Eichhornia crassipes*) were also collected from each site for stable isotope analysis (SIA). Concurrent water level data were provided by the Lake Naivasha Riparian Association.

Muscle samples for SIA were taken from crayfish in 2001, 2002, 2003, 2006, 2007 and 2008 and from carp in 2003 (when they first appeared in gill-net samples), 2006, 2007 and 2008. Individuals selected for SIA were sub-sampled from a uniform adult size range (Carp: 200 to 600 mm fork length; Crayfish: 40 to 55 mm carapace length) with a consistent annual mean to ensure inter-annual consistency. Concurrently, we sampled all the abundant basal resources and primary consumers to establish whether inter-annual variation in crayfish and carp isotope values were a result of changes in diet rather than changes in the stable isotope ratios of putative food resources. Ensuring consistency in the isotope values of basal resources over time is especially important when using such a metric approach. All samples were processed on an annual basis to avoid any degradation of tissue. SIA was performed at Queen Mary, University of London following the protocols of Ings et al. [26]. Ratios of ^15^N:^14^N and ^13^C:^12^C are expressed using conventional delta notations (δ) relative to international standards (sucrose for carbon; ammonium sulphate for nitrogen; see Ings et al. [26]).

### Mixing models

We used the Bayesian mixing model SIAR [27] to provide an estimate of the relative contribution of various resources assimilated by crayfish and carp. This model integrates variability in resource and consumer isotope values, providing a distinct advantage over other mixing models. Separate mixing models were run for each year for both carp and crayfish using available food resources, including chironomids, oligochaetes, submerged and floating plants, plant debris, benthic fine particulate organic matter (FPOM), and hippo dung. Hippos are pseudo-ruminants and produce large quantities of partially fermented dung; the Naivasha population are conservatively estimated to introduce ∼5800 tonnes of dung to the lake per annum [28]. In addition, crayfish was included as a resource for carp. These resources were the only ones to be sampled in sufficient abundance for stable isotope analysis and gut content analysis revealed their occurrence in the diet of both study species (Jackson, *pers. obs*). Fractionation factors between resources and the consumers were assumed to be 2.3±0.28‰ for δ^15^N and 0.4±0.17‰ for δ^13^C, based on a meta-analysis by McCutchan et al. [29].

### Population metrics

We used five quantitative population metrics derived from stable isotope data to reveal key aspects of trophic structure. The metrics were adapted from community–level metrics developed originally by Layman et al. [12] based on the mean δ^13^C and δ^15^N of all species in a community. We used the stable isotope values from all individuals sampled in these calculations, resulting in final metric values encompassing intra-population variation in diet. Additionally, all metrics were bootstrapped (*n* = 10000; indicated with a subscript ‘_b_’) based on the minimum sample size in the data set (*n* = 15) to allow comparison among populations among years because sample size varied. The metric *mean distance to centroid* (CD_b_) was used as a measure of population trophic diversity. CD_b_ is calculated as the mean Euclidean distance of each individual of a population to the δ^15^N-δ^13^C centroid for that population. The population metrics *nitrogen range* (NR_b_) and *carbon range* (CR_b_) correspond to the distance between the two individuals with the highest and lowest δ^13^C and δ^15^N values within a population and provide an indication of the total nitrogen and carbon range exploited by a population [12]. The metric *standard deviation of nearest neighbour distance* (SDNND_b_) can be used to infer population trophic evenness. SDNND_b_ is calculated as the standard deviation of Euclidean distances of each individual to its nearest neighbour in stable isotope bi-plot space. The community metric *total area* (TA) can be converted directly to a measure of population niche area [14]. However, Layman et al. [14] calculated TA from a convex hull drawn around the most extreme data points on an isotope bi-plot. This will give an incomparable measure of niche area when applied to different sample sizes (such as those used in our study; *n* = 15–89) since the convex hull area will generally increase with sample size even if the underlying population has remained the same [17]. Consequently, we use standard ellipse area (SEA) as a measure of the mean core population isotopic niche which is robust to variation in sample size, although we acknowledge that a convex hull better emphasises the role of individuals in the overall dispersion within isotope niche space [17]. Briefly, the standard ellipse is to bivariate data as standard deviation is to univariate data. The standard ellipse of a set of bivariate data is calculated from the variance and covariance of the *x* and *y* data and contains approximately 40% of the data [30], [31] and hence, it reveals the core niche area and is expected to be insensitive to sample size. However, the use of a (n-2) correction on the denominator in place of the standard (n-1) when calculating variances seems appropriate given the loss of an extra degree of freedom involved when dealing with bivariate data. Indeed, as supported by extensive simulation studies [17], a sample size corrected version of the standard ellipse area, referred to as SEA_c_ is employed here to circumvent the bias that arises when sample sizes are small. Explicitly,
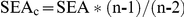
This correction has the property of increasing SEA_c_ at small sample sizes in order to correct bias towards underestimation but asymptotes to 1 at infinity. Furthermore, the calculation of SEA_c_ allows the degree of isotopic niche overlap to be calculated which can be then used as a quantitative measure of dietary similarity among populations. These methods, developed by Jackson et al. [17], are the first to provide quantitative measures of a population's trophic ecology that account for variation in sample size and correct for small sample sizes. All metrics were calculated using the R statistical computing package (R Development Core Team, 2007), see Jackson et al. [17] for detailed methodology and Layman et al. [12] for original descriptions of the community-level metrics.

Finally, we quantified annual changes in the reliance of crayfish on different resources to elucidate alterations in annual diet following the invasion of carp. We calculated the Euclidean distance between the mean crayfish isotope values for each consecutive year sampled and quantified the angle of change between subsequent mean crayfish isotope signatures. A vector-diagram was used to illustrate the changes [9]. Angles of change allow diet shifts to be distinguished from trivial annual fluctuations in species mean isotope values, while the distance of change will indicate the magnitude of any diet shifts.

All data were tested for normality and heteroscedasticity using Kolmogorov-Smirnov and Levene's tests, respectively (in Minitab® 14; Minitab Ltd., Pennsylvania, USA) before further statistical tests. Submerged-plant data failed these assumptions and were log_10_(x+1)-transformed. We tested for differences in δ^13^C and δ^15^N between species and among years with permutational analysis of variance [32], [33] using the PERMANOVA+ add-in to PRIMER® version 6.1 (PRIMER-E Ltd, Plymouth, UK). This was done with Type III sums of squares and was based on a Euclidian distance matrix and 9999 permutations of the residuals under a reduced model.

## Results

### Temporal changes

**Figure 1:**
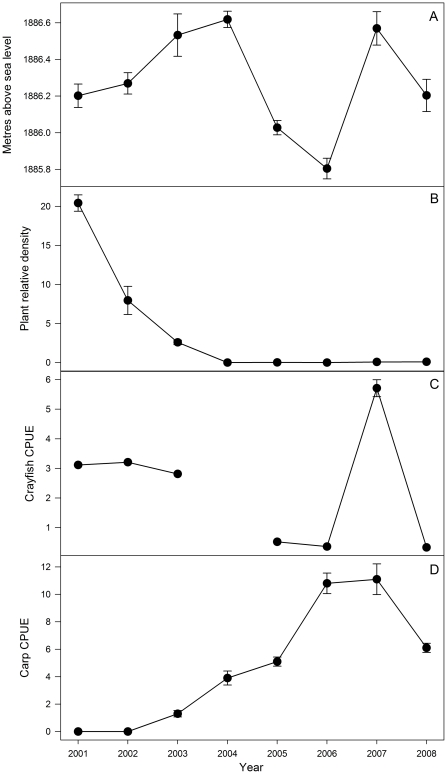
Mean (± standard error) annual water level (A), submerged plant relative abundance (B), crayfish CPUE (C) and carp CPUE (D) from 2001 to 2008.

Following their establishment, carp CPUE increased rapidly and consistently every year until 2008, when their abundance declined by 50% compared with 2007 (Fig. 1). There was no correlation between carp and crayfish abundance and water level (carp *r_7_* = −0.22, *P* = 0.6; crayfish *r_7_* = 0.38, *P* = 0.4). Submerged plant relative abundance was correlated inversely with carp abundance (*r_7_* = −0.77, *P* = 0.02) but not with crayfish (*r_7_* = 0.35, *P* = 0.44).

**Figure 2:**
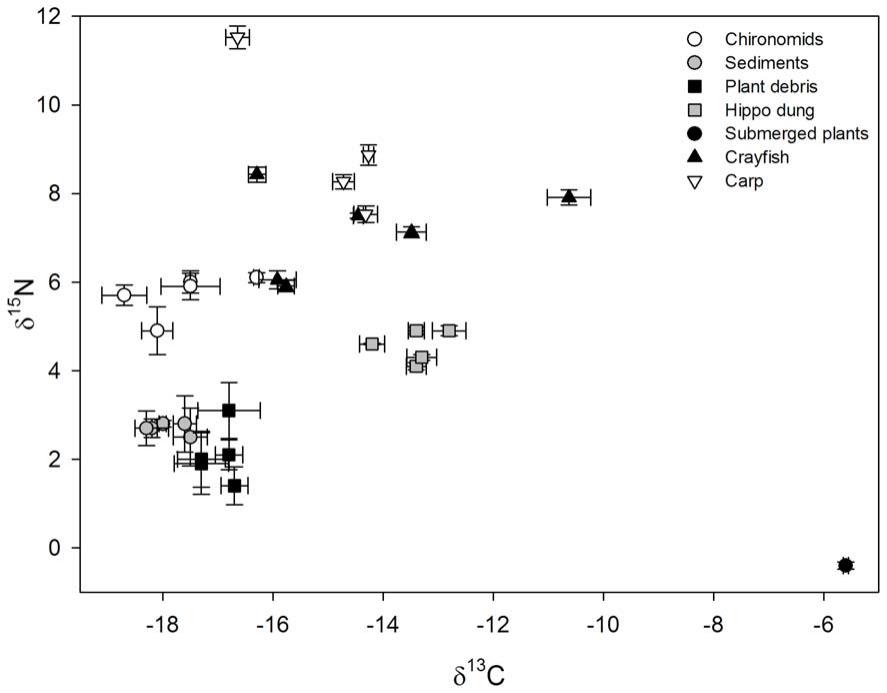
Stable isotope bi-plot showing the intra- and inter-annual variation in isotope values of resources, carp and crayfish. Each data point represents an annual mean and the error bars represent the intra-annual standard error.

The stable isotope values of basal resources and primary consumers remained consistent throughout the whole period of study (see Fig. 2) with no significant changes in either δ^13^C (permutational ANOVA; *F*_4,81_ = 2.10, *P* = 0.10) or δ^15^N (permutational ANOVA; *F*_4,81_ = 1.59, *P* = 0.19; Table 1) among years. In contrast, stable isotope values of carp and crayfish varied considerably among years (see below) when compared to the low variability in putative resources (Fig. 2) and therefore, we attribute any changes in the stable isotope values and population metrics of crayfish and carp to actual diet alteration.

**Table 1:** δ^13^C and δ^15^N values for basal resources and dominant benthic primary consumers.

Resource	Year	δ^13^C	δ ^15^N	n
Chironomids	2001	−18.7±0.7	5.7±0.4	3
	2002	−18.1±0.6	4.9±1.2	5
	2003	−17.5±0.2	6.0±0.5	5
	2006	−16.3±0.1	6.1±0.2	5
	2007	−17.5±1.2	5.9±0.7	5
Oligochaetes	2001	−16.0±0.3	3.5±0.5	3
	2002	−17.5±0.4	4.5±0.7	3
	2007	−16.1±1.0	5.1±0.1	2
Sediments	2001	−18.0±0.1	2.8±0.1	3
	2002	−17.5±0.6	2.5±1	4
	2003	−18.2±0.5	2.7±0.4	3
	2006	−17.6±0.4	2.8±1.1	3
	2007	−18.3±0.3	2.7±0.7	3
Plant debris	2001	−16.8±1.0	3.1±1.1	3
	2002	−16.7±0.4	1.4±0.7	3
	2003	−17.3±0.8	1.9±1.2	3
	2006	−16.8±0.4	2.1±0.6	3
	2007	−17.3±0.7	2.0±1.1	3
Water hyacinth	2001	−24.5±0.2	3.3±0.2	3
	2003	−26.6±0.5	4.0±0.2	3
	2007	−24.9±0.3	3.9±1.0	3
Hippo dung	2001	−13.4±0.4	4.1±0.2	6
	2002	−12.8±0.6	4.9±0.2	4
	2003	−13.3±0.6	4.3±0.1	5
	2006	−13.4±0.4	4.9±0.2	7
	2007	14.2±0.5	4.6±0.04	6
Submerged plants	2001	−5.6±0.1	−0.4±0.2	9

Each value (mean ± standard error) is based on multiple sampling efforts (n) each year. Chironomid and oligochaete samples comprised >20 individuals each time.

In total, 114 carp and 346 crayfish were analysed for stable isotopes. We found significant interactions between species and year for both δ^13^C (permutational ANOVA; *F*_3,449_ = 4.38, *P* = 0.006) and δ^15^N (*F*_3,449_ = 8.37, *P* = 0.0002). However, δ^13^C did not differ between carp and crayfish in 2003 or 2006, but was significantly higher in carp relative to crayfish in both 2007 and 2008 (*p*<0.001 in both cases; Fig. 2). Carp δ^15^N was consistently significantly higher than that of crayfish (*p*≤0.0001 in each case; Fig. 2).

### Mixing models

Native submerged plants were only available as a resource in 2001 due to their cyclic relationship with crayfish abundance in Lake Naivasha [22]. In 2001, submerged plants were the second most important resource in the diet of crayfish after hippo dung, contributing an average of 30% (Table 2). Hippo dung also contributed the most to crayfish diet relative to other resources in 2002, 2006 and 2007. Chironomids were the second most important resource in crayfish diet in 2002 and 2006 and the most important in 2003. The contribution of water hyacinth and benthic FPOM to crayfish diet was negligible in most years (Table 2).

**Table 2:** The relative contribution of putative resources to the diet of crayfish and carp from 2001 to 2007.

Species	Year	Resource	Low 95% hdr	Mean % contribution	High 95% hdr
Crayfish	2001	Chironomids	0.00	0.10	0.25
		Oligochaetes	0.00	0.07	0.19
		Plant debris	0.00	0.05	0.13
		Benthic FPOM	0.00	0.03	0.09
		Water hyacinth	0.00	0.02	0.05
		Hippo dung	0.09	0.43	0.77
		Submerged plants	0.02	0.30	0.58
	2002	Chironomids	0.00	0.10	0.21
		Oligochaetes	0.00	0.09	0.21
		Plant debris	0.00	0.02	0.06
		Benthic FPOM	0.00	0.03	0.09
		Hippo dung	0.65	0.75	0.85
	2003	Chironomids	0.68	0.77	0.87
		Plant debris	0.00	0.02	0.04
		Benthic FPOM	0.00	0.02	0.05
		Water hyacinth	0.00	0.01	0.03
		Hippo dung	0.10	0.18	0.26
	2006	Chironomids	0.34	0.40	0.46
		Plant debris	0.00	0.04	0.08
		Benthic FPOM	0.00	0.04	0.09
		Hippo dung	0.46	0.52	0.58
	2007	Chironomids	0.00	0.05	0.13
		Oligochaetes	0.00	0.11	0.26
		Plant debris	0.12	0.30	0.47
		Benthic FPOM	0.00	0.12	0.30
		Water hyacinth	0.00	0.02	0.06
		Hippo dung	0.23	0.39	0.53
Carp	2003	Chironomids	0.00	0.12	0.32
		Plant debris	0.00	0.04	0.12
		Benthic FPOM	0.00	0.04	0.13
		Water hyacinth	0.00	0.05	0.10
		Hippo dung	0.00	0.05	0.15
		Crayfish	0.41	0.70	0.92
	2006	Chironomids	0.00	0.12	0.25
		Plant debris	0.00	0.04	0.11
		Benthic FPOM	0.00	0.04	0.10
		Hippo dung	0.05	0.19	0.33
		Crayfish	0.40	0.61	0.81
	2007	Chironomids	0.00	0.08	0.20
		Oligochaetes	0.00	0.10	0.26
		Plant debris	0.00	0.02	0.04
		Benthic FPOM	0.00	0.02	0.04
		Water hyacinth	0.00	0.01	0.03
		Hippo dung	0.03	0.26	0.48
		Crayfish	0.22	0.52	0.83

Estimated using Bayesian mixing models. Contributions are designated as estimated low 95% highest density region (hdr), mean contribution, and high 95% hdr.

There was little variation in the contribution of each resource to crayfish diet between 2001 and 2002 except that because the submerged plants disappeared, the relative proportion of hippo dung increased in 2002. Once carp had appeared in the fishery in 2003, the contribution of each resource to the diet of crayfish varied considerably among years (Table 2). There was an increase in the contribution of chironomids until 2007 when there was a large increase in the relative contribution of plant debris (up to 47%; Table 2); this was coincident with the highest recorded carp abundance in the lake (Fig. 1). Crayfish contributed at least 22% to the diet of carp relative to the other resources in all years analysed. Indeed, in 2003 the average contribution of crayfish to the diet of carp was 70%. Chironomids, Oligochaetes and hippo dung also contributed to the diet of carp, whereas the contribution of plant debris, water hyacinth and benthic FPOM was negligible (Table 2).

### Population metrics

**Figure 3:**
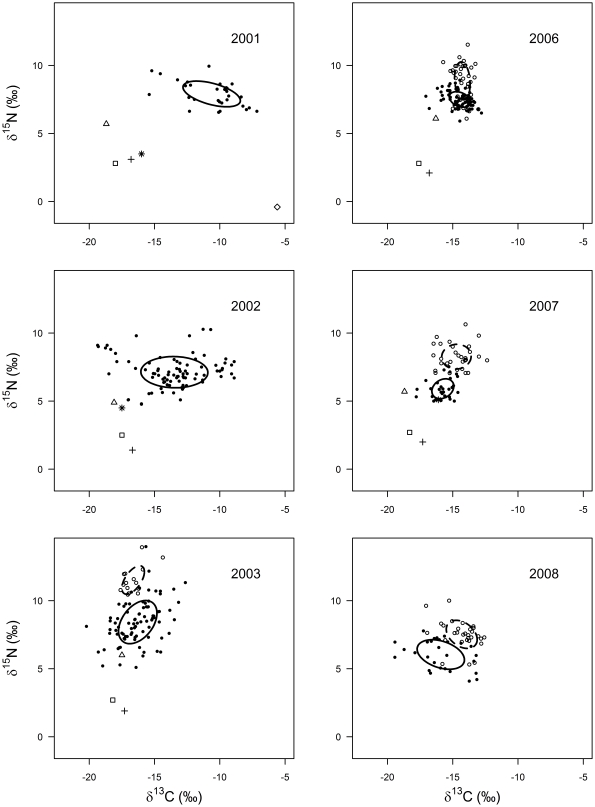
Stable isotope bi-plots for each year, illustrating the isotopic niche of carp and crayfish. The black circles represent individual crayfish and the open circles represent individual carp. The lines enclose the standard ellipse area (SEA_c_) for each year for both crayfish (solid) and carp (dashed). Mean values of resource points are also shown; benthic FPOM (open square), plant debris (cross), submerged plants (open diamond), chironomids (open triangle) and oligochaetes (asterisk).

**Figure 4:**
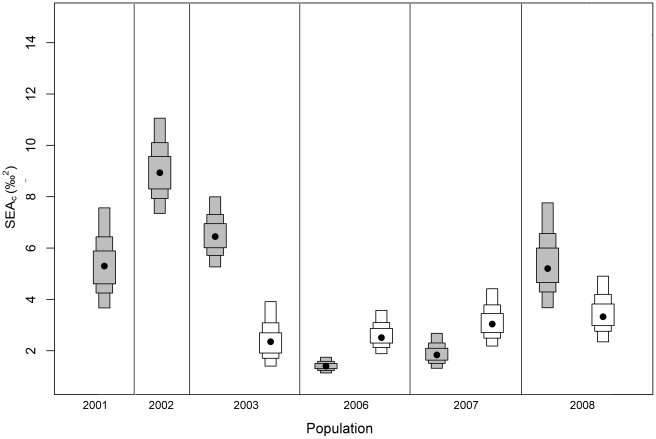
Density plot showing the confidence intervals of the standard ellipse areas. The black points correspond to the mean standard ellipse area for each group while the grey and white boxed areas reflect the 95, 75 and 50% confidence intervals for crayfish and carp, respectively.

The SEA_c_ of crayfish and carp did not overlap except in 2006 (Fig. 3) when crayfish were at very low abundance in the lake (Fig. 1). The area of overlap comprised 20.4% and 10.9% of total crayfish and carp isotopic niche area, respectively. The SEA_c_ of carp did not vary notably between years and remained relatively consistent in size, increasing slightly over the duration of the study (Fig. 3; Fig. 4). There was a positive correlation between carp SEA_c_ and the water level of the lake (*r*_5_ = 0.99, *P* = 0.04, Fig. 1). A higher water level could have improved resource diversity by providing access to terrestrial resources in the inundated zones.

**Figure 5:**
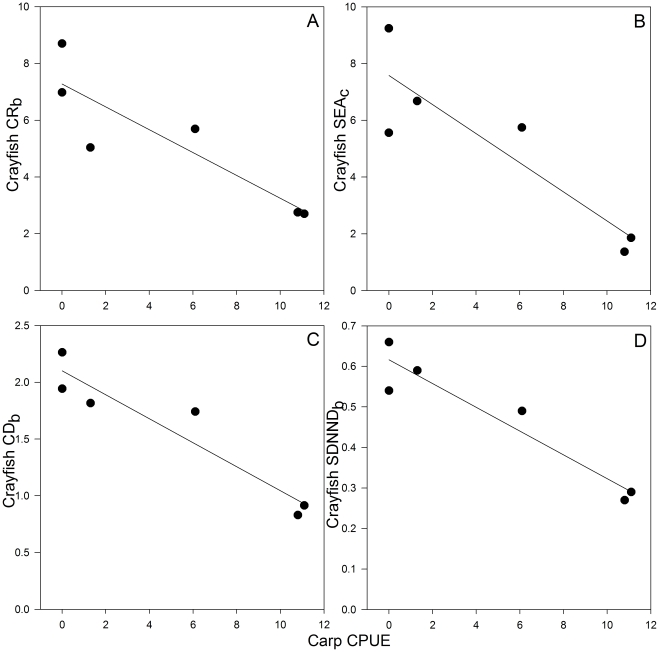
Relationships between carp CPUE and crayfish population metrics. A. carbon range (CR_b_), B. standard ellipse area (SEA_c_), C. mean distance to centroid (CD_b_) and D. standard deviation of nearest neighbour distance (SDNND_b_). Least-square regression lines are included for illustrative purposes only.

In contrast, the isotopic location of the crayfish SEA_c_ differed substantially among years and varied significantly in size (Fig. 3; Fig. 4). The SEA_c_ of crayfish decreased considerably from 2003, when carp were first found in low abundances in the lake, until 2006 and then increased slightly in 2007, by which time carp contributed to 90% of the commercial fishery catch (Fig. 1; Fig. 3; Fig 4). In 2008, the crayfish SEA_c_ increased by approximately three times, coinciding with a 50% reduction in carp CPUE (Fig. 1; Fig. 3). Additionally, crayfish exhibited their lowest NR_b_ and CR_b_ when carp catch was highest in 2006 and 2007 (Fig. 1; Fig. 3 and Table 3). In comparison, carp NR_b_ and CR_b_ remained similar in all years (Table 3). There was an inverse relationship between carp catch and both crayfish CR_b_ (*r*_5_ = −0.89, *P* = 0.02; Fig. 5A) and crayfish SEA_c_ (*r*_5_ = −0.89, *P* = 0.02; Fig. 5B).

**Table 3:** Population metrics for carp and crayfish in Lake Naivasha, grouped by year caught.

	NR_b_	CR_b_	CD_b_	SDNND_b_	SEA_c_
Carp 2003 (*n* = 15)	3.06	2.59	1.05	0.39	2.30
Carp 2006 (*n* = 37)	4.18	1.94	1.26	0.30	2.57
Carp 2007 (*n* = 31)	2.88	3.46	1.22	0.37	3.23
Carp 2008 (*n* = 30)	3.64	3.74	1.26	0.47	3.58
Crayfish 2001 (*n* = 30)	2.91	6.98	1.94	0.54	5.56
Crayfish 2002 (*n* = 89)	4.07	8.70	2.26	0.66	9.24
Crayfish 2003 (*n* = 85)	5.53	5.04	1.82	0.59	6.68
Crayfish 2006 (*n* = 84)	1.93	2.75	0.83	0.27	1.37
Crayfish 2007 (*n* = 30)	2.22	2.70	0.92	0.29	1.86
Crayfish 2008 (*n* = 28)	3.16	5.69	1.74	0.49	5.75

NR_b_ = δ^15^N range; CR_b_ = δ^13^C range; CD_b_ = mean distance to centroid; SDNND_b_ = standard deviation of mean nearest neighbor distance; SEA_c_ = standard ellipse area. The number of individuals used to calculate the metrics is shown in parentheses.

**Figure 6:**
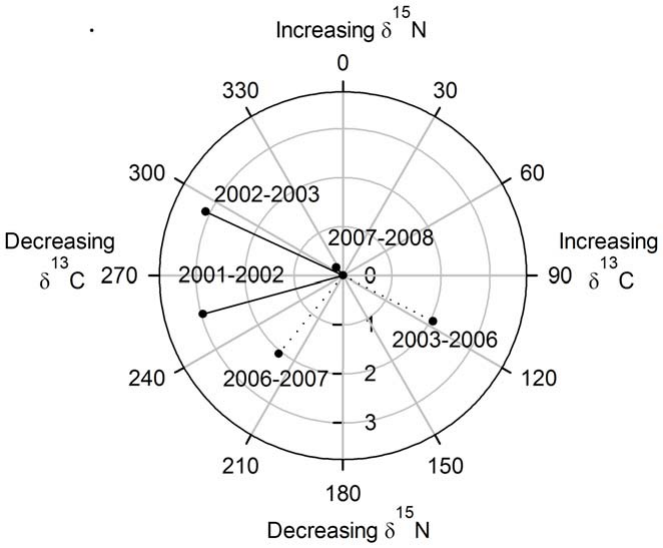
Arrow-diagrams showing the change in mean crayfish isotopic composition before and after carp dominance. Lines show the change before (solid lines) and after (dashed lines) carp dominance. Each arrow displays the mean isotopic change of crayfish carbon and nitrogen values compared to the previous sampling period. The length of each arrow represents the magnitude of change and the direction illustrates the angle of change.

The angle of change between each consecutive mean crayfish isotope value indicated an increasingly ^13^C-depleted diet (Fig. 6) with one exception (2003 to 2006). This was in parallel to the decline in availability of submerged plants which had high δ^13^C values (−5.6±0.1‰). Once carp had become established as the dominant fish species, crayfish exhibited angles of change reflecting a shift in diet towards lower trophic levels (*i.e.* reduced δ^15^N, Fig. 6). The only exception to this pattern occurred between 2007 and 2008 when the mean crayfish δ^15^N increased, coinciding with a 50% decline in carp CPUE (Fig. 1). The magnitude of change in crayfish diet was greatest between 2002 and 2003 (Fig. 6), coincident with carp first appearing in the fishery (Fig. 1).

The diversity of crayfish diet, measured as the *mean distance to centroid* (CD_b_) was lowest in 2006 and 2007 (Table 3) coinciding with the highest relative abundance of carp (Fig. 1). Further, there was a significant inverse correlation between crayfish CD_b_ and carp abundance (CPUE: *r*_5_ = −0.95, *P* = 0.004; Fig. 5C), and a significant inverse correlation between carp abundance and crayfish standard deviation of mean nearest neighbour distance (SDNND_b_; *r*_5_ = −0.95, *P* = 0.003; Fig. 5D), a measure of the spread of individuals within isotopic space. Crayfish abundance was not, however, correlated significantly with any carp isotopic population metrics (NR_b_: *r*_5_ = −0.42, *P* = 0.40; CR_b_: *r*_5_ = −0.08, *P* = 0.87; CD_b_: *r*_5_ = −0.26, *P* = 0.62; SDNND_b_: *r*_5_ = −0.24, *P* = 0.65; SEA_c_: *r*_5_ = −0.20; *P* = 0.7).

## Discussion

Our stable isotope-derived metrics of trophic structure provide novel ways of quantifying interactions among populations and/or species and their application revealed new insights into interactions between two globally widespread sympatric invasive species. There was a considerable reduction in crayfish isotopic niche (measured as SEA_c_) following establishment of carp, another benthic omnivore, thus supporting our hypothesis that carp would suppress (and/or displace) the isotopic niche of crayfish. The isotopic niche of crayfish subsequently increased in size when they appear to have been largely released from interspecific competition in 2008 as carp abundance declined sharply, presumed to be due to heavy fishing pressure in the commercial fishery. The angle and magnitude of annual change in mean crayfish isotope values indicated that there was also displacement of the crayfish niche following carp establishment. This isotopic niche shift throughout the study was directed away from a previously important resource; native submerged plants [22].

Following the carp population expansion (2003 to 2006), crayfish also exhibited an isotopic change toward a lower trophic position, suggesting a shift in diet to avoid dietary overlap and subsequent competition with carp. The only deviation from this trend was between 2007 and 2008 when crayfish shifted toward a slightly higher trophic position, coincident with a 50% reduction in carp CPUE. The relative contribution (shown by Bayesian mixing models) of putative food resources to the diet of crayfish altered after carp establishment, supporting our hypothesis that the location of the dietary niche of crayfish would change following the carp invasion. Chironomids and hippo dung contributed to the diet of crayfish in all years, whereas the relative contribution of plant debris was below 10% except in 2007. This exception coincided with the highest measure of carp abundance, when crayfish may have been forced to feed on a lower quality resource as a result of competition or carp habitat alteration. Furthermore, our stable isotope-derived population metrics suggest that there was an important interaction occurring between the two invaders; high carp abundance reduced the diversity of crayfish diet (measured as CD_b_), increased packing of individuals in isotopic space (measured as SDNND_b_), limited the total range of exploited resources (measured as CR_b_), and reduced the number of trophic levels utilised (measured as NR_b_). In contrast, the abundance of crayfish did not affect any isotopic metrics of carp, indicating that crayfish presence had little impact on carp trophic ecology. Hence, the interaction among crayfish and carp was asymmetric, with carp altering crayfish trophic ecology and not vice versa.

Asymmetric competition may have arisen due to the larger size carp attain, which can provide competitive superiority [34]. Carp and crayfish were isotopically distinct in all years except 2006 when the core dietary niche of carp and crayfish overlapped and when carp abundance was reduced by the commercial fishery. The majority of niche partitioning in all other years was due to higher δ^15^N values of carp, implying a higher trophic position. Crayfish was the most important assimilated resource for carp relative to the other resources in all years analysed (shown using Bayesian mixing models) and this is supported by the analyses of carp gut contents [23]. This suggests that intraguild predation, whereby a superior predator (i.e. carp) both feeds on and competes for resources with another species (i.e. crayfish), played a role in the decline in crayfish abundance [35]. We observed a reduction in crayfish trophic diversity at times of high carp abundance when there was no isotopic niche overlap (measured using SEA_c_) in the majority of years, suggesting that the two invaders also interacted indirectly, possibly due to behavioural modifications or competition for non-food resources.

On three recent sampling occasions (Jul and Nov 2009, March 2010), we failed to trap any crayfish and, on the basis of our isotope data, it may imply that the interaction with carp has led to the virtual elimination of crayfish in Lake Naivasha. Serial replacement of invasive species is an alternative theory to that of the Invasion Meltdown scenario [6]. Indeed, invasive carp also reduce crayfish abundance in their native habitat, which is attributed to carp-induced habitat depletion [36]. Common carp feed in the benthic zone which can uproot macrophytes [37]. An inverse correlation between carp CPUE and the relative abundance of native submerged plants, an important food source for crayfish in Lake Naivasha [22], indicates possible habitat alteration by carp in the lake. Furthermore, a decline in plant abundance will also reduce the number of macroinvertebrates associated with those plants and may thus be responsible for the decline in the dietary niche area of crayfish. The mean annual change of crayfish isotope values was directed away from exploitation of submerged plants, reflecting the decline in their availability.

A diverse diet range and/or variation in resource use among individual crayfish from 2001 to 2003 (prior to carp dominating the fishery) was illustrated by substantial variability in isotope values and hence a high trophic diversity (measured as CD_b_) and large spread over isotopic space (measured as SDNND_b_). We infer, therefore, that the variety of available resources declined as a result of carp-induced habitat alteration from 2006 onwards, thus forcing the crayfish to exploit less diverse prey items which resulted in reduced isotopic variability and significantly reduced CD_b_, SEA_c_, CR_b_ and SDNND_b_ values [14]. The predation risk posed by carp may have altered crayfish foraging behaviour and use of refuge and, hence, caused a change in resource choice [38]. The alternative scenario, that increased competition for resources would result in a more varied diet to maintain energy requirements, was rejected. Despite the reduction in crayfish abundance, Lake Naivasha has shown no measurable ecological recovery (primarily in terms of submerged plants), suggesting that carp has replaced crayfish as a keystone species. Our study therefore highlights the dynamic nature of highly invaded ecosystems and indicates that the virtual elimination of crayfish from the lake will likely force carp to utilise other resources. This, in turn, suggests that there will soon be another significant shift in the food web structure of Lake Naivasha.

A combination of stable isotope derived-population metrics and Bayesian mixing models revealed a complex interaction between invasive carp and crayfish in Lake Naivasha. The detrimental impact carp had on the crayfish population appears to have been due to a number of dietary interactions, including predation [23] and indirect dietary interactions mediated via habitat depletion. We were able to draw these conclusions from the population metrics due to the consistency in the isotopic composition of each resource throughout the study, signifying that the changes in crayfish niche size and position were a consequence of changes in the proportion and/or identity of assimilated resources. This conclusion is supported further by the results of the Bayesian mixing model, SIAR. It is important to consider fluctuations in resource isotopic composition, since the isotopic area occupied by putative resources will directly influence the isotopic area occupied by consumers. It is also important to consider that the metric SEA_c_ quantifies the core comparable isotopic niche of a species or population and hence, the community metric TA [12] may be more applicable to some analyses if the full isotopic area occupied by the species/population is required.

The population metrics calculated from stable isotope data proved a useful tool to reveal subtle dietary interactions between species and demonstrated potential for application to a wide range of fields in ecology. The increasing pace of global environmental change has had substantial impacts on local biodiversity and it is imperative to understand those ecological interactions ultimately responsible for the patterns observed. The community metrics developed by Layman et al. [12] caused some controversy and discussion when first published [39] but ultimately added another ecological tool-set to help unravel the complexity of food webs. Through providing a logical extension to their approach, such population-level metrics can now be used widely in conjunction with appropriate measures of ecosystem structure and functioning to reveal the direct and indirect consequences of local environmental change on populations.
